# Heterogeneity in Comparisons of Discontinuation of Tumor Necrosis Factor Antagonists in Rheumatoid Arthritis - A Meta-Analysis

**DOI:** 10.1371/journal.pone.0168005

**Published:** 2016-12-08

**Authors:** Anat Fisher, Ken Bassett, Gautam Goel, Dana Stanely, M. Alan Brookhart, Hugh R. Freeman, James M. Wright, Colin R. Dormuth

**Affiliations:** 1 Department of Anesthesiology, Pharmacology & Therapeutics, University of British Columbia, Vancouver, British Columbia, Canada; 2 Department of Family Practice, University of British Columbia, Vancouver, British Columbia, Canada; 3 Department of Family and Community Medicine, University of Toronto, Toronto, Ontario, Canada; 4 Department of Epidemiology, Gillings School of Global Public Health, University of North Carolina, Chapel Hill, North Carolina, United States of America; 5 Department of Medicine, University of British Columbia, Vancouver, British Columbia, Canada; Saint George's University, UNITED KINGDOM

## Abstract

**Objective:**

We did a systematic review of studies comparing discontinuation of tumor necrosis factor alpha (TNF) antagonists in rheumatoid arthritis (RA) patients, pooled hazard ratios and assessed clinical and methodological heterogeneity.

**Methods:**

We searched MEDLINE and EMBASE until June 2015 for pairwise hazard ratios for discontinuing infliximab, etanercept, and adalimumab from cohorts of RA patients. Hazard ratios were pooled using inverse variance weighting and random effects estimates of the combined hazard ratio were obtained. Clinical and methodological heterogeneity was assessed using the between-subgroup I-square statistics and meta-regression.

**Results:**

Twenty-four unique studies were eligible and large heterogeneity (I-square statistics > 50%) was observed in all comparisons. Type of data, location, and order of treatment (first or second line) modified the magnitude and direction of discontinuation comparing infliximab with either adalimumab or etanercept; however, some heterogeneity remained. No effect modifier was identified when adalimumab and etanercept were compared.

**Conclusion:**

Heterogeneity in studies comparing discontinuation of TNF antagonists in RA is partially explained by type of data, location, and order of treatment. Pooling hazard ratios for discontinuing TNF antagonists is inappropriate because largely unexplained heterogeneity was demonstrated when random effect estimates were calculated.

## Introduction

The tumor necrosis factor alpha (TNF) antagonists target a cytokine that regulates inflammation in multiple diseases, including rheumatoid arthritis (RA) [1]. Evidence on the relative efficacy and safety of these medications is indirect and incomplete because no randomized controlled trials (RCTs) directly compare two or more TNF antagonists in RA patients [2]. Lack of efficacy and adverse effects are the most common reasons for discontinuing TNF antagonists [3–9], and therefore discontinuation risk is a good measure of the benefit-harm balance of these medications [10]. Hence, comparison of discontinuation risk of different TNF antagonists can help in treatment decisions, especially selection of an individual medication.

Since their introduction in the late 1990s, multiple observational studies have compared discontinuation of TNF antagonists, but the results were inconsistent [11–15] due to methodological and clinical heterogeneity. Methodological heterogeneity, defined as “variability in study design and risk of bias” [16], may be caused, for example, by differences in data collection. Clinical heterogeneity, defined as “variability in the participants, interventions and outcomes” [16], could be caused by differences in location and dates, or frequency of dose adjustments. A previous systematic review summarized hazard ratios for discontinuing TNF antagonists but failed to identify predictors of methodological or clinical heterogeneity [15]. The objective of this study is to investigate methodological and clinical heterogeneity in hazard ratios for discontinuing TNF antagonists in RA patients.

## Methods

### Systematic literature search

Electronic databases (MEDLINE and EMBASE) to June 2015 were searched using the following strategy: (1) adalimumab.mp. (2) infliximab.mp (3) etanercept.mp. (4) tumour necrosis factor antagonists.mp. or Receptors, Tumour Necrosis Factor/ (5) 1 or 2 or 3 or 4 (6) (patient compliance or adherence or persistence or discontinuation or switching or treatment duration).mp. [mp = ti, ab, sh, hw, tn, ot, dm, mf, ps, rs, nm, ui] (7) rheumatoid arthritis.mp. or rheumatoid arthritis/ (8) 5 and 6 and 7. Additional studies were identified by reviewing reference lists of publications meeting the inclusion criteria and other published reviews.

### Selection criteria for studies

We included studies of RA patients treated with infliximab, adalimumab, or etanercept that met the following criteria:

#### Study design

Cohort studies with multiple TNF antagonists. RCTs were excluded due to differences between RA patients in RCTs and those treated in routine clinical practice [17–20]. Studies were selected regardless of the language and the type of publication (full articles, abstracts, or conference proceedings).

#### Participants

RA patients, based on either the American College of Rheumatology diagnosis criteria [21,22] or the clinical judgment of the care-providing physicians. Studies of multiple diseases were included only if the outcomes of interest were presented separately for RA.

#### Types of interventions

First or second line treatments with infliximab, adalimumab, or etanercept selected by the care-providing physician and/or the patient. Studies of the newer TNF antagonists, such as certolizumab pegol or golimumab, were excluded due to shorter availability and fewer studies [15].

#### Duration of follow-up

At least one year from treatment initiation.

#### Outcome of interest

Pairwise hazard ratios for discontinuation: infliximab vs. etanercept, infliximab vs. adalimumab, and adalimumab vs. etanercept.

### Data extraction

Two reviewers (AF and GG/DS) independently selected studies and extracted data. In case of a discrepancy, a decision was reached by consensus. Authors of published studies were contacted when reports were incomplete, confusing, or difficult to interpret. The reviewers extracted as-reported hazard ratios, and 95% confidence intervals (CI) or p-value. If the hazard ratio for a specific comparison was missing, we attempted to calculate it using indirect comparison methodology [23] or synthesis of estimates from subgroups. To prevent the use of duplicate or overlapping data from the same source, we selected a single hazard ratio from a fully-published manuscript with the largest population for each comparison and data source.

### Risk of bias

We identify two specific sources of bias in studies of discontinuation and included only studies with low risk of bias, defined as:

1. The study outcome was discontinuing the individual medication or switching to a second biologic anti-rheumatic medication. Patients remaining on treatment at the end of the study period were censored.
2. Discontinuation was not associated with the likelihood to be included in the study; i.e., new-user design without mandatory minimum treatment duration. In prevalent-user design, patients who started treatment before the study period are included only if they are still treated at the beginning of the study; hence, patients with longer use are overrepresented.

### Statistical analysis

Hazard ratios for discontinuation with 95% CI were combined using an inverse variance approach, and data were recorded on the natural logarithm scale [24]. We calculated random effect estimates [25] because substantial heterogeneity has previously been observed [11,15].

In the absence of a definitive statistical test to assess whether a factor causes heterogeneity, we identified effect modifiers. We tested for the association between the effect size and clinical factors: continent, order of treatment, age, sex, and Disease Activity Score (DAS-28) as well as methodological factors: type of data and duration of follow-up. Categorical factors consisted of continent (Europe, Asia, or America), order of treatment (first or second line), and type of data (clinical charts, disease or drug registries, or administrative claim data). For these factors, we conducted between-subgroup I-square statistics, and estimated the significance using chi-squared test [26]. For continuous factors, i.e., age, sex, baseline DAS-28, and duration of follow-up, we conducted meta-regression [27] with a fixed effect model and weights based on the inverse of the variance of the logarithm of the hazard ratio. For factors that were reported as the average or the median of populations, we stratified the regression model by type of central measure. A significant association between a factor and the effect size was defined as a two-tailed p-value <0.05 for both categorical and continuous variables. Analyses were conducted using the Review Manager (RevMan) statistical software (Version 5.3, The Nordic Cochrane Centre, The Cochrane Collaboration, Denmark) and SAS software package (Version 9.4, SAS Institute Inc., Cary, NC).

## Results

**Fig 1:**
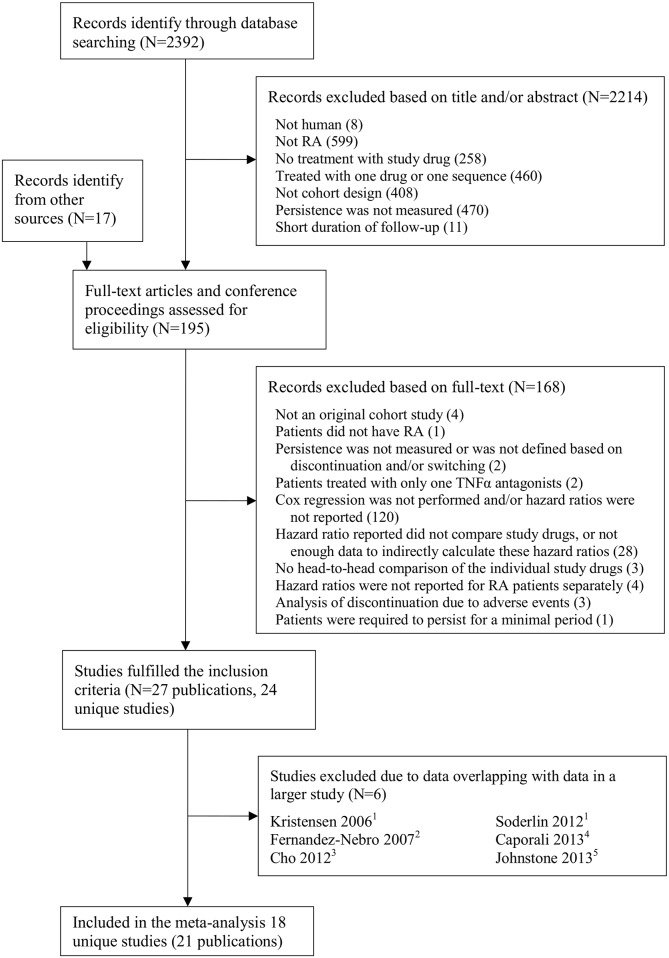
QUOROM flow chart. ^1^ SSATG is part of ARTIS, data were overlapping with Neovius 2015. ^2^ Carlos Haya hospital is included in BIOBADASER 2.0, data were overlapping with Gomez-Reino 2012. ^3^ Data from South Korea NIH, also known as Health Insurance Review and Assessment Service, were included in Lee 2014. ^4^ Data from MonitorNet were included in Scire 2013. ^5^ Data from MarketScan were included in Johnstone 2015.

A total of 2,409 unique citations were identified and screened (Fig 1), and 24 unique studies were eligible for inclusion (Table 1). Forty studies reported hazard ratios for discontinuing TNF antagonists but were excluded, most commonly because the study drugs were not compared (S1 Table in the on-line supporting information). Two of the studies were excluded due to high risk of bias [28,29]. Three studies reported outcomes from the SSTAG/ARTIS Swedish registry [5,30,31], two studies from the Spanish BIOBADASER 2.0 registry or hospitals contributing to it [6,32], two studies from the Italian Monitornet registry [33,34], two studies from the American claim database MarketScan [35,36], and three studies from the national insurance claim data or hospitals in South Korea [37,38] (Table 1).

**Table 1:** Characteristics of eligible studies.

Reference	Data source	Period	RA diagnosis	Type of users	Previous DMARDs	Persistence/ discontinuation	N (INF, ADA, ETA)	Follow up
Kristensen 2006 [39]	South Swedish Arthritis Treatment Group (SSATG), Sweden	March 1999—December 2004	Clinical judgement by the treating physician. 98% fulfilled the ACR 1987 criteria	Biologics naive	≥2, including MTX previously without satisfactory response	Registered prospectively, based on the judgement of the treating physician.	1161 (721, 440)	Not reported
Fernandez-Nebro 2007 [32]	A tertiary care center, a structured clinical follow-up protocol, Spain	March 1999—January 2006	ACR criteria	Anti-TNF-naive	≥2, including MTX previously without satisfactory response	"Definitive"	161 (60, 22^a^, 79)	Mean (STD) 20.6 (16.8) months; range 0.0–62.2; median 24
Borah 2009 [40]	Claims data (I3 Innovus), a large managed health care plan, US	January 2005—December 2006	≥1 medical claim with RA as the primary diagnosis prior to the index date	≥6 months without dispensing	Not reported	>30-day medication-free gap or switching	1230 (0, 527, 703)	12 months
Du Pan 2009 [41]	Swiss Clinical Quality Management for Rheumatoid Arthritis (SCQM-RA) registry, Switzerland	January 1997—December 2006	Not reported	78% anti-TNF naive	Not reported	> 6 month medication-free gap	2364 (595, 882, 887)	Not reported
Marchesoni 2009 [42]	LORHEN registry, Italy	January 1999 –December 2001	ACR criteria	First course in the registry	≥1 course of combination therapy, one of which should always be MTX without satisfactory response	Discontinuation due to clinical remission—censored	1064 (519, 303, 242)	6–36 months of follow-up, or discontinued therapy within 6 months
Hetland 2010 [43]	DANBIO registry, Denmark	October 2000–3 April 2009	clinical judgement by the treating physician	Not reported	≥1 without satisfactory response	Not reported	2326 (1134, 675, 517)	Median (IQR) for adalimumab, 20 months 7–39); etanercept, 21 months (9–42); infliximab, 16 months (5–36)
Cho 2012 [37]	National Health Insurance (NHI) claim database, South Korea	January 2007—December 2009	A diagnosis of RA (ICD10-M05 or M06)	New-user design (washout period from January 2007 to June 2007 without anti-TNF)	Not reported	>14-week refill free gap Persistence = the number of days between the first and last refills	388(26^b^, 219, 143)	Not reported
Gomez-Reino 2012 [6]	BIOBADASER 2.0, Spain	February 2000 –December 2010	Not reported	First treatment	Not reported	Not reported	2097 (1273, 761, 873)	First year
Greenberg 2012, [44]	CORRONA registry, US	February 2002—March 2008	Not reported	(1) Biologics naive (2) First time switchers	Not reported	Data was collected every 3 months. " we used the visit dates of reported initiation and visit dates of reported discontinuation"	(1) 1475 (535, 460,480) (2) 616 (166, 311, 139)	Not reported
Soderlin 2012 [31]	South Swedish Arthritis Treatment Group (SSATG) biologics register, Sweden	March 1999—December 2005	A clinical diagnosis	First anti-TNF course	MTX alone or in combination without any satisfactory response and/or intolerance	Not reported	534	A minimum of 3.6 years
Caporali 2013 [33] [A]	Monitornet database, Italy	from January 2007	Not reported	First course	Not reported	Not reported	1992(426, 685, 881)	Not reported
Chen 2013 [45] [A]	National Health Insurance (NHI), Taiwan	Not reported	Not reported	Anti-TNF naïve	Not reported	>84-day refill free gap	4592 (0, 1982,2609)	First year
Hishitani 2013 [46]	Osaka BiRD registry, Japan	September 1999—April 2012	ACR criteria	Biologics naive^c^	≥1	Discontinuation due to remission or miscellaneous reasons and missing data were treated as censored cases	401^d^ (103, 58, 143)	Not reported
Johnston 2013 [35] [A]	Truven Health MarketScan databases, US	January 2010—June 2011	Not reported	Used at least one biologic prior to index	Not reported	A 90-day medication-free gap or switching to another biologic	7515^e^ (672, 1504, 1114)	Not reported
Scire 2013 [34]	Monitornet database, Italy	January 2007—April 2012	Not reported	Anti-TNF-naive	Failure	Medication interruption ≥ 3 months. Persistence = the number of days between the first and last day of treatment	2640 (718, 887, 1035)	Not reported
Senabre-Gallego 2013 [47,48] [A]	"our local cohort" Asociación para la Investigación en Reumatología de la Marina Baixa (AIRE-MB), Spain	January 2001—November 2011	Not reported	Not reported	Not reported	Not reported	318 (97, 116, 105)	Not reported
Fisher 2014 [49]	BC Ministry of Health databases, Canada	March 2001—December 2009	≥2 outpatient at least 60 days apart or ≥1 inpatient diagnosis of RA (ICD-9 714.XX) the three years prior to TNF-b initiation	Biologics naive	Not reported	≥180 days medication-free gap or switching to another ‘biologic’	2286 (620, 344, 1322)	Not reported
Flouri 2014 [50]	Hellenic Registry of Biologic Therapies, Greece	January 2004—April 2011	according to the treating physician	79% anti-TNF naïve	≥1	"registered prospectively"	1028 patients, 1297 courses (560, 435, 302)	The median (IQR) 3.0(1.2–6.2) years for infliximab, 2.9(1.1–5.9) years for adalimumab, and 2.9(1.1–5.0) years for etanercept.
Frazier-Mironer 2014 [51,52]	Medical charts, eight rheumatology centers, France	March 2005—April 2011	1987 ACR criteria	(1) Biologics naïve (2) second anti-TNF medication	Not reported	The first definitive treatment interruption or last observation on treatment after initiation (exact time collected via the patient chart): indicated by the treating rheumatologist, or no consecutive re-introduction of treatment	(1) 706 (99, 203, 404) (2) 231 (20^f^, 105, 106)	2–6 years
Kang 2014 [53,54]	Medical charts, Chonnam National University Hospital, Gwangju, South Korea	December 2002—November 2011	ACR criteria	Anti-TNF naive	Not reported	Not reported	144 (22, 48, 39)	At least one year
Lee 2014 [38] [A]	Health Insurance Review and Assessment Service, South Korea	2006—December 2010	≥2 prescriptions of DMARD under the diagnosis of RA	New-user design (washout period without DMARDs during 2006)	Not reported	Medication-free gap of > half of the days supply of the previous prescription, or switching to other TNF inhibitors	2203 (458, 1202, 543)	Not reported
Neovius 2015, [5]	Swedish Biologics Register (ARTIS), Sweden	January 2003—December 2011	Assessment of the treating rheumatologists	Anti-TNF-naive	Not reported	As reported by the treating rheumatologist, due to any cause, except for pregnancy and remission. discontinuation	2898	Up to 5 years
Johnston 2015 [55]	Truven Health MarketScan database, US	January 2010 –December 2011	ICD-9-CM codes recorded on medical claims between January 2009 and March 2012	Previously used ≥1 other biologic	Not reported	≥90 days Medication-free gap or switching to another biologic	9782^g^ (922, 2179, 16750	Not reported

^a^ Patients treated with adalimumab were excluded due to the reduced sample size.

^b^ Patients treated with infliximab were excluded from analysis, since this medication was not available throughout the analysis period

^c^ Patients who started therapy before entering the registry were included.

^d^ Including 97 patients treated with tocilizumab.

^e^ Including patients treated with abatacept (1297), certolizumab (681), golimumab (951), and rituximab (622).

^f^ Patients treated with infliximab were excluded from analysis of second anti-TNF due to small numbers.

^g^ Including patients treated with abatacept (1759), certolizumab (962), golimumab (1195), and tocilizumab (1090)

[A] Abstract, ACR American Collage of Rheumatology, DMARDs Disease Modifying Anti-Rheumatic Drugs, ICD-9 the International Classification of Diseases Ninth Revision, ICD-9-CM the International Classification of Diseases Ninth Revision Clinical Modification, ICD10 the International Classification of Diseases Tenth Revision, IQR Interquartile Range, MTX Methotrexate, STD Standard Deviation, TNF Tumor Necrosis Factor alpha, US United Stated of America

Fifteen studies (20,796 patients) from unique data sources compared infliximab and adalimumab with the overall pooled hazard ratio of 1.08 (95% C] 0.92–1.27) (S1 Fig). Fifteen studies (23,671 patients) from unique data sources compared infliximab and etanercept with the overall pooled hazard ratio of 1.22 (1.00–1.49) (S2 Fig). Seventeen studies (27,799 patients) from different data sources showed higher risk of discontinuing adalimumab compared with etanercept and the overall pooled hazard ratio was 1.17 (1.08–1.27) (S3 Fig). There was significant heterogeneity between studies for all three comparisons, with I square statistics of 86%, 92%, and 56%, respectively.

**Fig 2:**
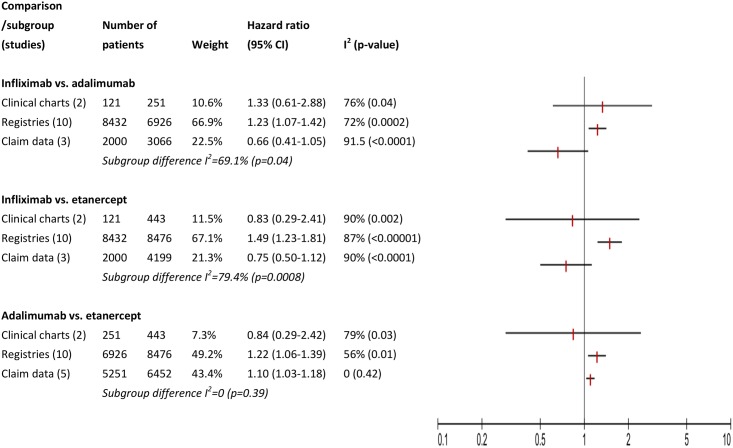
Assessment of methodological heterogeneity: subgroup analysis for type of data.

**Fig 3:**
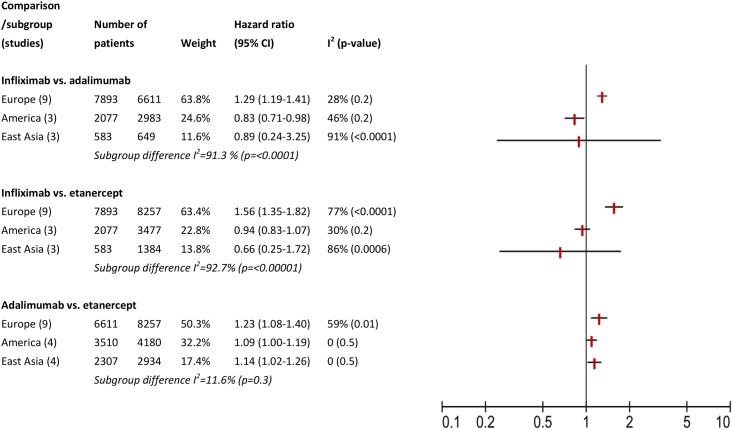
Assessment of clinical heterogeneity: subgroup analysis for location.

**Fig 4:**
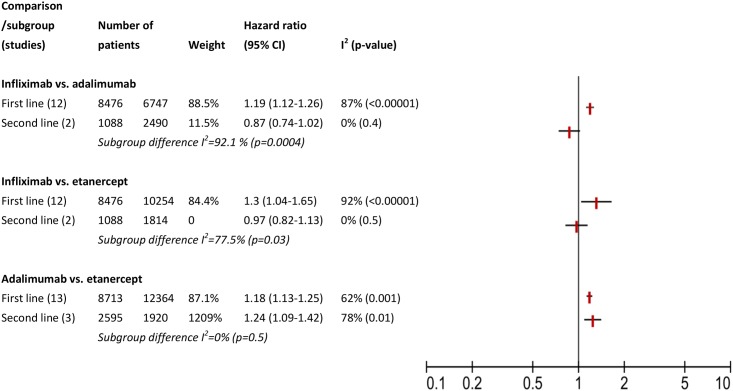
Assessment of clinical heterogeneity: subgroup analysis for order of treatment.

Assessment of methodological and clinical heterogeneity is presented in Table 2. In analysis of categorical factors, effect modifications of the type of data (Fig 2), location (Fig 3), and order of treatment (Fig 4) was observed in comparisons of infliximab with adalimumab or etanercept, but not comparing adalimumab with etanercept. This effect modification was expressed as I squared statistics of 69.1–92.7%, with p-value <0.05 in Chi squared test. These percentages could be interested as following: 69.1–92.7% of variation across subgroups in each comparison is due to heterogeneity rather than chance. We also noticed that in all comparisons, not all subgroup hazard ratios reach statistical significance level and in most cases a residual within subgroup heterogeneity was observed. For example, in analysis of type of data (Fig 2), when comparing infliximab with etanercept, we observed significant heterogeneity between the three subgroups compared: studies based on clinical charts, those conducted on registries and analyses of claim data (I square statistics of 69.1%). Only studies conducted on registries had a significant pooled hazard ratio of 1.49 (95% CI 1.23–1.81), but they also consisted the largest subgroup. A reversed direction of hazard ratio was estimated in two studies based on clinical charts and three analyses of claim data, i.e., lower risk of discontinuing infliximab, but these polled estimates did not reach significance level. We noticed residual heterogeneity within each subgroup: clinical chart, registries, and claim data.

**Table 2:** Assessment of heterogeneity: association between study design and patient characteristics and effect sizes.

	Factor tested and statistics	Infliximab vs. adalimumab	Infliximab vs. etanercept	Adalimumab vs. etanercept
Clinical heterogeneity	Continent I^2^, p-value	82.7, <0.0001	91.3, <0.0001	0, 039
	Order of treatment I^2^, p-value	77.5, 0.03	92.1, 0.004	0, 0.49
	Age (infliximab users), regression parameter (standard error), p-value	0.015 (0.032), 0.66	0.006 (0.029), 0.8	
	Age (adalimumab users), regression parameter (standard error), p-value	0.037 (0.034), 0.30	n/a	0.008 (0.022), 0.72
	Age (etanercept users), regression parameter (standard error), p-value	n/a	0.142 (0.085), 0.14	-0.006 (0.022), 0.78
	Sex (infliximab users), regression parameter (standard error), p-value	0.77 (0.532), 0.18	4.668 (1.49), 0.01	n/a
	Sex (adalimumab users), regression parameter (standard error), p-value	0.757 (0.443), 0.12	n/a	-0.140 (0.398), 0.73
	Sex (etanercept users), regression parameter (standard error), p-value	n/a	2.054 (0.929), 0.05	-0.186 (0.486), 0.71
	Baseline DAS (infliximab users), regression parameter (standard error), p-value	0.055 (0.084), 0.54	-0.234 (0.338), 0.51	n/a
	Baseline DAS (adalimumab users), regression parameter (standard error), p-value	0.051 (0.072), 0.51	n/a	-0.088 (0.165), 0.61
	Baseline DAS (etanercept users), regression parameter (standard error), p-value	n/a	-0.225 (0.266), 0.43	-0.145 (0.193), 0.48
Methodological heterogeneity	Type of data I^2^, p-value	79.4, 0.008	69.1, 0.04	11.6, 0.32
	Duration of follow-up, regression parameter per 10 years (standard error), p-value	-0.004 (0.001), 0.62	-0.033 (0.03), 0.30	-0.001 (0.004), 0.90

P-value <0.05 represents a significant effect of the factor tested on the hazard ratio in the individual comparison (between-subgroup I-square statistics [26] and p-value of chi-squared test for categorical factors, and meta-regression [27] with a fixed effect model and weights based on the inverse of the variance of the logarithm of the hazard ratio for continuous factors).

DAS- disease activity score; n/a–not applicable

In analysis of continuous factors (Table 2), the proportion of female patients using infliximab modified the hazard ratio in comparison of infliximab with etanercept. However, in the presence of multiple comparisons and in the absence of similar effect of the proportion of female patients using etanercept we discarded this finding. Finally, duration of follow up, age, and baseline DAS-28 did not modify the hazard ratios (Table 2).

## Discussion

This review explored sources of clinical and methodological heterogeneity in studies comparing discontinuation of TNF antagonists in RA patients. The type of data (i.e. charts, registries, or claims) modified the effect size in comparisons of infliximab with etanercept or adalimumab. However, this factor was not responsible for all the heterogeneity. Different types of data are susceptible to different types of biases. Registries are susceptible to selection bias caused by the volunteer enrollment and data collection [56]. Administrative data are susceptible to confounding due to the absence of clinical variables and exposure ascertainment bias because of the uncertainty whether patients who refilled the medication actually used it. Type of data also determines how the outcome, discontinuation, is defined. In analysis of registries or medical charts, discontinuation is recorded by physicians, either during a routine visit or in real-time. In analysis of administrative data, discontinuation is usually ascertained using prescription-refill analysis and applying grace periods [57]. Comparisons of discontinuing TNF antagonists are especially sensitive to these differences in outcome definition because of the intermittent dosing schedules and different lengths of dose interval for different medications. Comparisons of infliximab were more sensitive to the data source probably because it has a significantly longer dose interval than adalimumab and etanercept.

A second hazard modifier is location. In European countries, the risk of discontinuing etanercept and adalimumab is lower compared to infliximab, but in America, patients on infliximab had lower discontinuation risk compared to adalimumab and similar risk as patients treated with etanercept. In a previous review reported similar proportions of patients from European and non-European countries who discontinued any TNF antagonists [15], but the results were not presented separately for each individual medication. Souto et al [15] failed to determine whether these findings are constant across different medications.

Hazard ratio estimates were also modified by the order of treatment (first or second line) in comparisons of infliximab with adalimumab or etanercept. However, in these comparisons the only two studies that reported hazard ratios for second line treatment were American studies Therefore, we cannot rule out that the modification observed is related to location and not to order of treatment.

Age, sex, baseline disease activity score (DAS), and duration of follow-up did not modify the hazard ratios. The absence of modification by baseline DAS opposes the hypothesis by Greenberg 2014 [58] that the difference in estimates between American and European studies is caused by differences in disease severity.

The results of this review question the reliability of hazard ratios for discontinuing TNF antagonists. Specifically, the residual heterogeneity within subgroups may indicate that stable results cannot be duplicated by different researchers nor can conclusive scientific findings be obtained. Alternately, researchers may not be measuring the same outcome because different types of data, and possibly different definitions of discontinuation, modified the hazard ratios. Standardization of methodological approaches may help achieving the requisite reliability.

There are several limitations to our study. First, we were unable to adequately assess risk of bias in the absence of a specific evaluation tool for discontinuation studies. Available tools for observational studies, such as Newcastle-Ottawa scale [59], do not assess relevant items such as new-user design and ascertainment of discontinuation. The other tools, e.g., STROBE statement [60], assess the quality of reporting and not the risk of bias. Second, in the absence of a statistical test to determine causes of heterogeneity between studies, we could only assess effect modification. Last, we found significant residual heterogeneity within many of the subgroups and therefore pooled estimates were impossible to interpret.

This review had several strengths including the wide scope: no temporal or linguistic constraints. Second, to minimize bias, this review included only studies reporting adjusted hazard ratios for discontinuation. Earlier systematic reviews summarized proportions of discontinuation for each TNF antagonist individually [14,15]. Because these proportions were crude estimates from observational data, comparisons between medications were most likely confounded. Last, we identified two major risks of bias in discontinuation studies and applied them in study selection.

## Conclusions

Substantial heterogeneity was found in studies estimating head-to-head hazard ratios for discontinuing TNF antagonists in RA patients due to differences in type of data, location, and order of treatment. The heterogeneity observed shows that stable results have not been duplicated by different researchers and conclusive scientific findings cannot be obtained by pooling results.

## Supporting Information

S1 Fig Forest Plots: Hazard Ratios of included studies: Infliximab vs. Adalimumab. (TIFF)

S2 Fig Forest Plots: Hazard Ratios of included studies: Infliximab vs. Etanercept. (TIFF)

S3 Fig Forest Plots: Hazard Ratios of included studies: Adalimumab vs. Etanercept. (TIFF)

S1 Table Excluded studies. (PDF)

S2 Table PRISMA 2009 checklist. (PDF)
