# Correction: Heterogeneity in Comparisons of Discontinuation of Tumor Necrosis Factor Antagonists in Rheumatoid Arthritis—A Meta-Analysis

**DOI:** 10.1371/journal.pone.0172646

**Published:** 2017-02-15

**Authors:** Anat Fisher, Ken Bassett, Gautam Goel, Dana Stanely, M. Alan Brookhart, Hugh J. Freeman, James M. Wright, Colin R. Dormuth

The sixth author’s name is spelled incorrectly. The correct name is: Hugh J. Freeman. The correct citation is: Fisher A, Bassett K, Goel G, Stanely D, Brookhart MA, Freeman HJ, et al. (2016) Heterogeneity in Comparisons of Discontinuation of Tumor Necrosis Factor Antagonists in Rheumatoid Arthritis—A Meta-Analysis. PLoS ONE 11(12): e0168005. doi:10.1371/journal.pone.0168005
