# Supplementary figures and images for: Heterogeneity in Comparisons of Discontinuation of Tumor Necrosis Factor Antagonists in Rheumatoid Arthritis - A Meta-Analysis
Source: PLoS One. 2016 Dec 8;11(12):e0168005. doi: 10.1371/journal.pone.0168005 (PMC5145210; doi:10.1371/journal.pone.0168005)

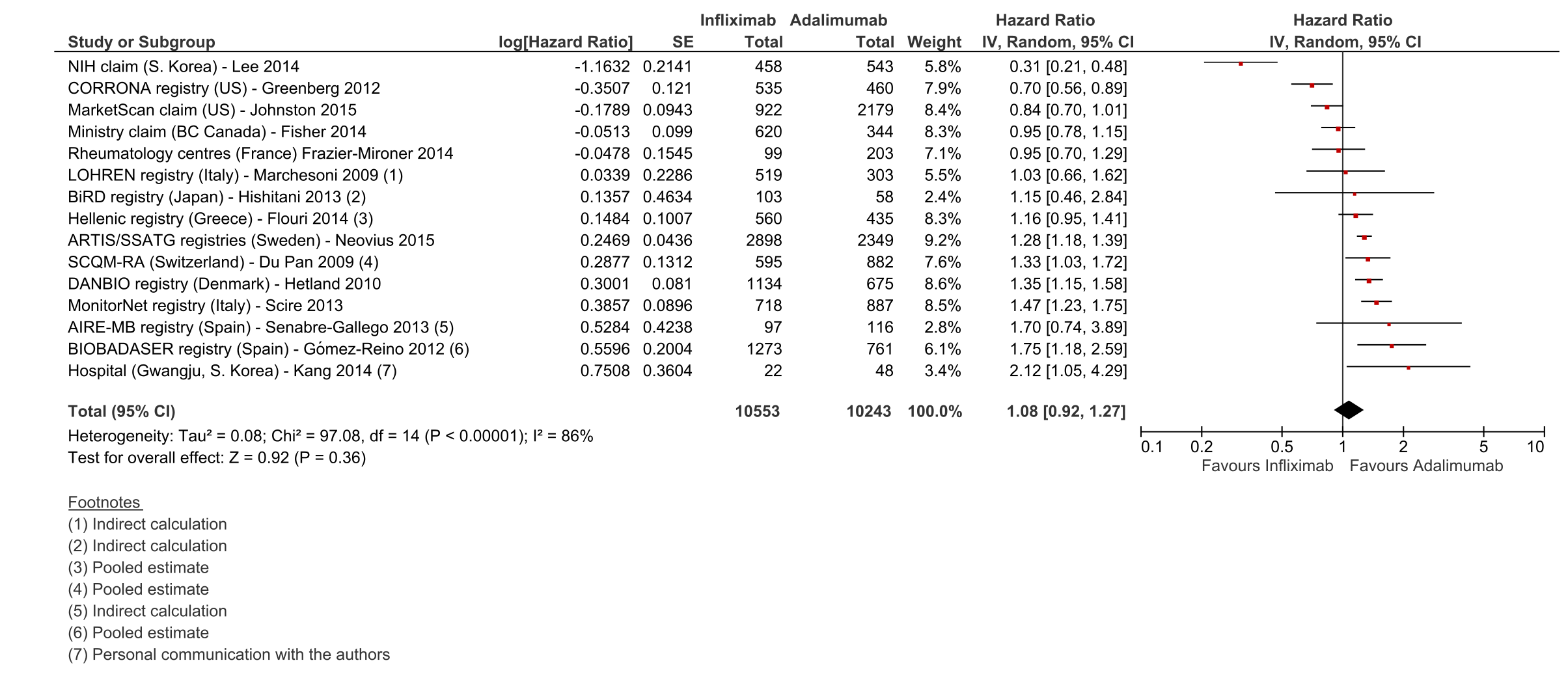

Supplement: S1 Fig — (TIFF) [file pone.0168005.s001.tiff]

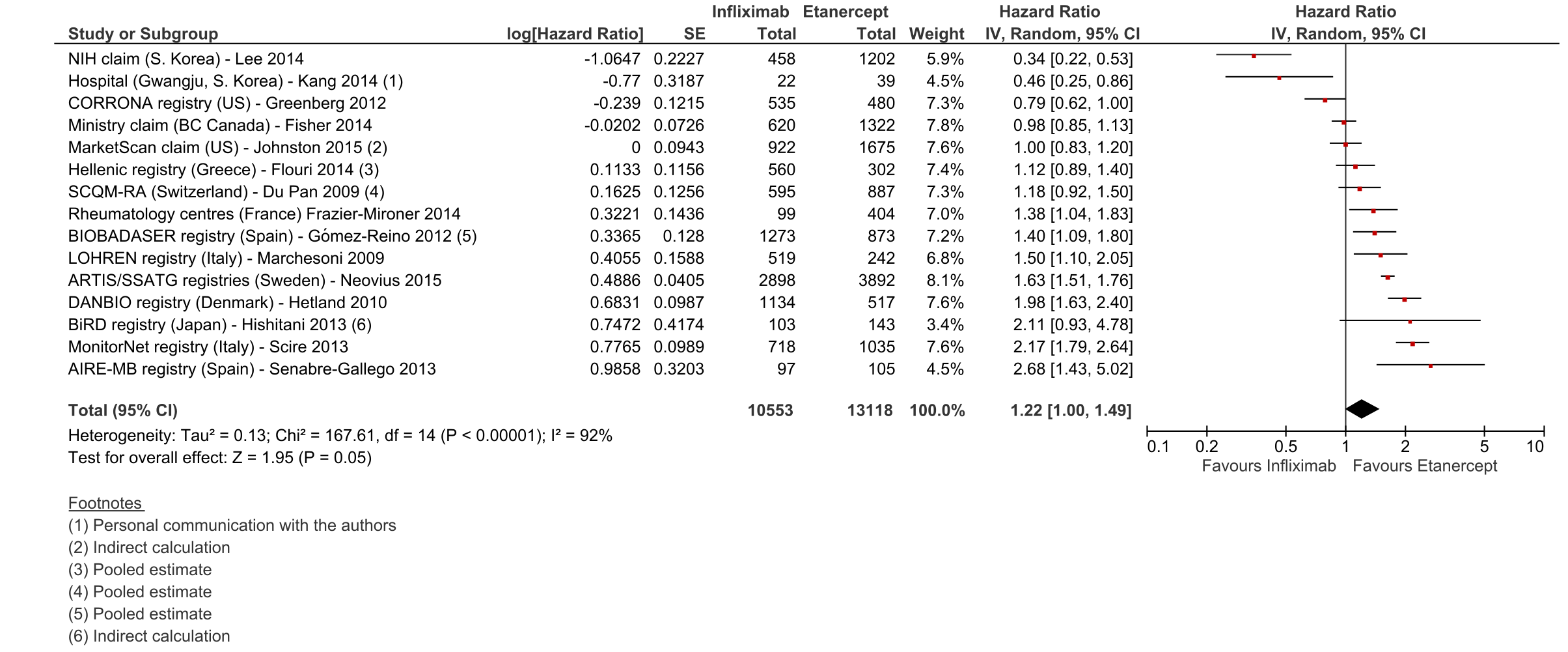

Supplement: S2 Fig — (TIFF) [file pone.0168005.s002.tiff]

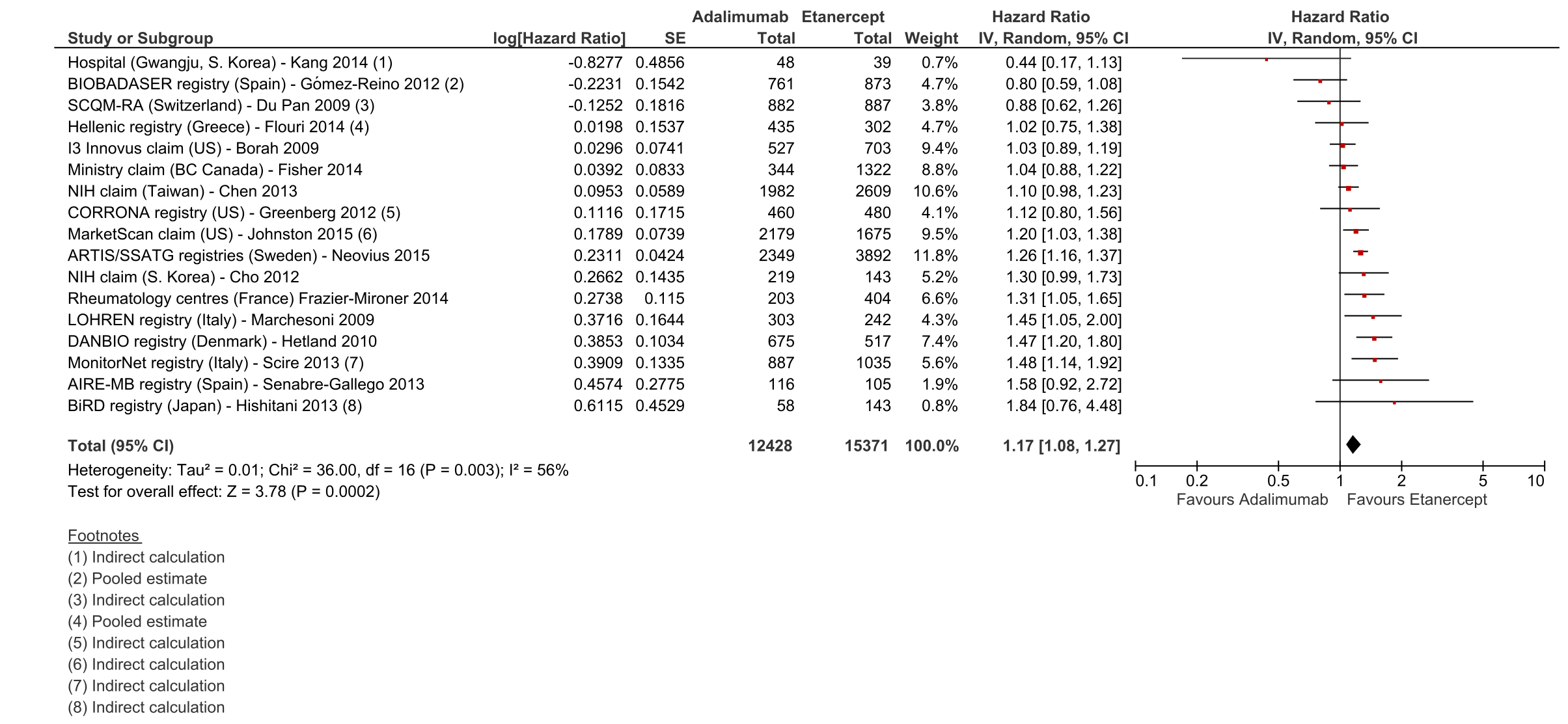

Supplement: S3 Fig — (TIFF) [file pone.0168005.s003.tiff]
