# Supplementary material for: Heterogeneity in Comparisons of Discontinuation of Tumor Necrosis Factor Antagonists in Rheumatoid Arthritis - A Meta-Analysis
Source: PLoS One. 2016 Dec 8;11(12):e0168005. doi: 10.1371/journal.pone.0168005 (PMC5145210; doi:10.1371/journal.pone.0168005)
Supplement: S1 Table — (PDF) [file pone.0168005.s004.pdf]

Reasons	Studies excluded
Hazard ratio reported did not compare study medications, or not enough data to indirectly calculate these hazard ratios	1. Alvaro-Gracia, Ann Rheum Dis 2013;72<br>2. Cho Ann Rheum Dis 2014;73.<br>3. Choquette Arthritis and Rheumatology 2014;66:S216.<br>4. Du Pan Ann Rheum Dis 2012;71(6):997-999.<br>5. Fafa Clin Rheumatol 2015;34(5):921-927.<br>6. Favalli Rheumatology (United Kingdom) 2014;53(9):1664-1668<br>7. Gabay Ann Rheum Dis 2013;72.<br>8. Gulfe J Rheumatol 2009;36(3):517-521.<br>9. Kojima Modern Rheumatology 2012;22(3):339-345.<br>10. Markenson J Rheumatol 2011;38(7):1273-1281.<br>11. Ogale BMC Musculoskeletal Disorders 2011;12:204.<br>12. Sakai Arthritis Res Ther 2015;17(1):74-015-0583-8.<br>13. Soliman Ann Rheum Dis 2011;70(4):583-589.<br>14. Tunceli Arthritis Rheum 2012;64:S1118.<br>15. Yoshida Arthritis Rheum 2011;63 (10 SUPPL. 1.<br>16. Zhang Pharmacoepidemiol Drug Saf 2013;22:22.<br>17. Zhang Arthritis Rheum 2013 October 2013;65:S990-S991.<br>18. Agarwal J Rheumatol 2008;35(9):1737-1744.<br>19. Curkendall Arthritis Rheum 2008;59(10):1519-1526.<br>20. Descalzo Reumatologia Clinica 2007;3(1):4-20.<br>21. Doherty Rheumatology 2009;48:145.<br>22. Filippini Clinical Reviews in Allergy and

	Immunology 2010;38(2-3):90-96.<br>23. Finckh Ann Rheum Dis 2009;68(1):33-39.<br>24. Grijalva Med Care 2007;45(10 SUPPL. 2):S66-S76.<br>25. Hyrich J Rheumatol 2010;37(10):2021-2024.<br>26. Mattey J Rheumatol 2010;37(10):2021-2024.<br>27. Soliman Rheumatology 201;49:3-4.<br>28. Staples Rheumatology 2011;50(1):166-175.
No head-to-head comparison of the individual study medications	1. Finckh Ann Rheum Dis 2006;65(6):746-752.<br>2. Humby Arthritis and Rheumatology 2014;66:S43-S44.<br>3. Kobayakawa Modern Rheumatology 2015;25(2):251-256.
Hazard ratios were not reported for RA patients separately	1. Carmona Arthritis Res Ther 2006;8(3):R72.<br>2. Duclos J Rheumatol 2006;33(12):2433-2438.<br>3. Gomez-Reino Arthritis Res Ther 2006;8(1):R29.<br>4. Heiberg Arthritis Care and Research 2008;59(2):234-240.
Analysis of discontinuation due to adverse events	1. Abasolo Semin Arthritis Rheum 2015;44(5):506-513.<br>2. Rodriguez-Rodriguez Arthritis Rheum 2013;65:S1012.<br>3. Sakai Ann Rheum Dis 2012;71(11):1820-1826.
**High risk of bias** : Patients were required to persist for a minimal period	Zhang Arthritis Care Res (Hoboken) 2015;67(5):624-632.
**High risk of bias** : Prevalent cases selected during the identification period were included	Martinez-Santana Patient Preference and Adherence 2013;7:719-727
